# Acute L-Citrulline Supplementation Increases Nitric Oxide Bioavailability but Not Inspiratory Muscle Oxygenation and Respiratory Performance

**DOI:** 10.3390/nu13103311

**Published:** 2021-09-22

**Authors:** Anastasios A. Theodorou, Panagiotis T. Zinelis, Vassiliki J. Malliou, Panagiotis N. Chatzinikolaou, Nikos V. Margaritelis, Dimitris Mandalidis, Nickos D. Geladas, Vassilis Paschalis

**Affiliations:** 1 Department of Life Sciences, School of Sciences, European University Cyprus, Nicosia 1516, Cyprus; 2 School of Physical Education and Sport Science, National and Kapodistrian University of Athens, 17237 Athens, Greece; zinelis56@gmail.com (P.T.Z.); bmalliou@phed.uoa.gr (V.J.M.); dmndldis@phed.uoa.gr (D.M.); ngeladas@phed.uoa.gr (N.D.G.); vpaschalis@phed.uoa.gr (V.P.); 3 Department of Physical Education and Sport Science at Serres, Aristotle University of Thessaloniki, 61122 Serres, Greece; tso.p@hotmail.com (P.N.C.); nvmargar@auth.gr (N.V.M.); 4 Dialysis Unit, 424 General Military Hospital of Thessaloniki, 56429 Thessaloniki, Greece

**Keywords:** nitric oxide, L-citrulline, NIRS, respiratory muscles, sternocleidomastoid, ergogenic supplements, fatigue

## Abstract

The present study aimed to investigate whether acute L-citrulline supplementation would affect inspiratory muscle oxygenation and respiratory performance. Twelve healthy males received 6 g of L-citrulline or placebo in a double-blind crossover design. Pulmonary function (i.e., forced expired volume in 1 s, forced vital capacity and their ratio), maximal inspiratory pressure (MIP), fractional exhaled nitric oxide (NO^•^), and sternocleidomastoid muscle oxygenation were measured at baseline, one hour post supplementation, and after an incremental resistive breathing protocol to task failure of the respiratory muscles. The resistive breathing task consisted of 30 inspirations at 70% and 80% of MIP followed by continuous inspirations at 90% of MIP until task failure. Sternocleidomastoid muscle oxygenation was assessed using near-infrared spectroscopy. One-hour post-L-citrulline supplementation, exhaled NO^•^ was significantly increased (19.2%; *p* < 0.05), and this increase was preserved until the end of the resistive breathing (16.4%; *p* < 0.05). In contrast, no difference was observed in the placebo condition. Pulmonary function and MIP were not affected by the L-citrulline supplementation. During resistive breathing, sternocleidomastoid muscle oxygenation was significantly reduced, with no difference noted between the two supplementation conditions. In conclusion, a single ingestion of 6 g L-citrulline increased NO^•^ bioavailability but not the respiratory performance and inspiratory muscle oxygenation.

## 1. Introduction

L-citrulline is a nonessential, non-coded alpha-amino acid that has a key role during the urea cycle in the liver [1]. In the urea cycle, L-citrulline is synthesized from ornithine and metabolized by argininosuccinate synthetase [1]. During this cycle, there is no release of citrulline in the circulation, and also, hepatocytes are unable to uptake citrulline from the circulation [2,3]. Thus, citrulline synthesis in the liver is compartmentalized to the urea cycle and independent of the other metabolic pathways of citrulline. Until recently, it was considered that the primary precursor for citrulline synthesis was the consumption of L-glutamine. However, it appears that the contribution of L-glutamine to citrulline synthesis is minor [4]. Orally ingested L-citrulline mainly leads to the biosynthesis of L-arginine. L-citrulline, released from the small intestine into the circulation, bypasses hepatic metabolism and is absorbed by the proximal tubular cells of the kidneys [3]. Then, citrulline is converted into arginosuccinate, which is then converted into arginine [1]. The increased arginine bioavailability in the circulation serves as a precursor for the formation of nitric oxide (NO^•^) [5]. L-citrulline can also be synthesized via the NO^•^ cycle. This formation of NO^•^ from arginine is catalyzed by nitric oxide synthase with citrulline release [1].

NO^•^ is a signaling molecule that has a vital role in regulating vasodilation, blood flow, and muscle oxygenation [6,7]. Briefly, it regulates vascular tone and blood flow by activating soluble guanylate cyclase in the vascular smooth muscle [8] and controls mitochondrial cellular respiration by inhibiting cytochrome c oxidase [9]. Indeed, the increase of blood flow and oxygen delivery to skeletal muscle during exercise is achieved by vasodilators such as NO^•^ formed locally in muscle tissue [10,11]. Thus, supplementation with NO^•^ precursors such as L-citrulline and L-arginine has been considered as an ergogenic aid [12,13,14,15]. Compared to L-arginine, evidence supports that oral L-citrulline supplementation is a more efficient intervention for increasing NO^•^ synthesis [5,16]. This is because L-citrulline bypasses hepatic metabolism [3] by inhibiting arginase enzymes [17].

During exercise, blood flow in skeletal muscles is increased to match exercise’s oxygen demands and remove metabolic by-products [18,19]. Physiologically, the increase in inspiratory and expiratory muscle work leads to an increased demand for blood flow and oxygen delivery to the respiratory muscles that must be sustained during exercise [20]. A mismatch between oxygen supply and demand increases the rate of fatigue development and decreases exercise performance [21,22]. More specifically, inspiratory muscle fatigue decreases exercise tolerance and impair working locomotor muscle performance [23,24,25,26]. This impairment in performance is probably due to reduced blood flow and oxygen supply to the inspiratory and locomotor muscles [27,28,29,30]. Even though several mechanisms have been implicated in regulating blood flow during exercise (e.g., increased cardiac output; vascular smooth muscle relaxation), NO^•^ appears to be the key molecule in regulating blood flow during exercise [31]. Hence, several NO^•^ precursors (e.g., L-citrulline, L-arginine) have been prescribed with the aim to increase resistance to fatigue and improve exercise performance by modulating blood flow and oxygen metabolism.

L-citrulline has received considerable scientific attention for its potential to increase NO^•^ bioavailability and improve exercise performance [15]. Increasing NO^•^ bioavailability during exercise with L-citrulline supplementation appears to induce greater tissue oxygenation, oxygen uptake [12,16], and peripheral vasodilation [32] in locomotor muscles. Thus, it is interesting to examine whether similar favorable changes in blood flow and oxygenation can diminish inspiratory muscles’ fatigability and enhance performance. Near-infrared spectroscopy (NIRS) is a non-invasive technique that continuously monitors regional tissue oxygenation in vivo. It has good sensitivity to detect and provide a reliable picture of muscle oxygenation changes during exercise [33,34]. Therefore, using the NIRS technique, this study aimed to investigate whether acute L-citrulline supplementation would affect sternocleidomastoid muscle oxygenation and respiratory performance. We chose to examine the sternocleidomastoid muscle since it is a crucial muscle for pressure generation during inspiration [35]. We hypothesized that an increase in NO^•^ synthesis would increase sternocleidomastoid muscle oxygenation and respiratory performance during resistive breathing to task failure.

## 2. Materials and Methods

### 2.1. Participants

Twelve recreationally trained healthy males [n = 12, mean  ±  standard deviation (SD): age =25 ± 5 yr, height = 178 ± 7 cm, body mass = 70.7 ± 6.7 kg, body mass index = 23.9 ± 1.2 kg/m^2^)] expressed interest to voluntarily participate in the study. Body mass was measured to the nearest 0.1 kg (Beam Balance 710, Seca, UK), with participants lightly dressed and barefoot. Standing height was measured to the nearest 1 cm (Stadiometer 208, Seca, UK). Participants were stable at their anthropometric characteristics for at least the last 2 years. The researchers asked the participants to recall whether they had participated in high-intensity resistance or aerobic training 2 weeks before the study entry. None of the subjects was a smoker or had any respiratory disease that could limit their ability to perform the resistive breathing sessions. Volunteers were instructed to abstain from any strenuous exercise during their participation in the study (except for the resistive breathing sessions performed during the experimental procedures). They were also asked to abstain from alcohol and caffeine consumption for 2 days before the assessments. Subjects did not receive medication/nutrient supplements known to influence the variables measured during their participation in the study. The researchers provided the volunteers with a written set of instructions for monitoring dietary consumption and a record sheet for recording food intake the 2 days before the first fatigue protocol. They were asked to follow the same food intake before the second fatigue protocol. Informed written consent was obtained for all participants after they were informed of all risks, discomforts, and benefits involved in the study.

### 2.2. Study Design

The study design used in the present investigation was counterbalanced crossover, double-blind, placebo controlled. Participants visited the laboratory on three different occasions. During the first visit, anthropometrical measurements, familiarization with the procedures, and evaluation of maximal inspiratory pressure using a portable spirometer (K5, Powerbreath, Southam, UK) were performed. One week after the first visit, subjects reported to the laboratory and performed the following baseline measurements: (i) pulmonary function via spirometry, (ii) fractional exhaled nitric oxide measurement in expiratory air, (iii) maximal inspiratory pressure, and (iv) inspiratory muscle oxygenation. After the baseline assessments, the subjects received 6 g of either L-citrulline (Now, L-Citrulline Pure Powder, Bloomingdale, IL, USA) or placebo (maltodextrin) in a double-blind, randomized crossover fashion. L-citrulline and maltodextrin powders were mixed with 150 mL of water in the proportions described above to produce the L-citrulline and placebo supplements, respectively. The weight of the supplementation was performed using an analytical balance (AES/AEJ, KERN & SOHN GmbH). An independent researcher performed simple randomization of the participants using Excel 365 (Microsoft, Redmond, WA, USA), and neither the participants nor the investigators were aware of the group assignment. According to the randomization schedule, a researcher who did not participate in the resistive breathing protocol and the assessments pre-packed L-citrulline and placebo in the same opaque bottles. L-citrulline and placebo were in a liquid form and identical in taste and appearance.

One hour after the supplementation, the participants repeated all the baseline measurements. Then, the participants performed an acute incremental resistive breathing to task failure of the respiratory muscles. The resistive breathing consisted of 30 inspirations of resistance corresponding to the 70% and 80% of maximal inspiratory pressure (30 inspirations at each resistance level) followed by continuous inspirations until exhaustion corresponding to the 90% maximal inspiratory pressure. Task failure was reached when the participants could not overcome the pressure applied by the apparatus. Immediately after the incremental resistive breathing to task failure, the participants repeated all the baseline measurements. The rate of perceived exertion was also assessed using the Borg scale. We chose to perform the resistive breathing protocol one hour after the supplementation based on published pharmacokinetic data [5]. The whole procedure was repeated after a two-week washout period in a crossover scenario. All data were unblinded for the statistical analysis after all procedures and assessments were completed.

### 2.3. Inspiratory Pressure

The assessment of maximal inspiratory pressure (MIP) was performed using a portable spirometer (K5, Powerbreath, Southam, UK), and the pressure was measured in mmH_2_O. During the acute resistive breathing, subjects had to inhale against a gradually increased inspiratory resistance until they could not overcome the pressure applied by the apparatus. The last successful effort before failure was considered as the maximal inspiratory pressure.

### 2.4. Pulmonary Function

The pulmonary function was assessed by measuring forced vital capacity (FVC), forced expiratory volume in 1 s (FEV_1_), and the FEV_1_/FVC ratio using a metabolic stress testing system (MedGraphics, CPX-D, Minnesota, MN, USA). The participants performed five regular breathing cycles in a seated position followed by a maximal inspiration and a 6 s maximal forced expiration. The expiration was a maximal effort and not tidal breathing. Three measurements were performed, and the best one was recorded and used for the statistical analysis.

### 2.5. Fractional Exhaled Nitric Oxide

Fractional exhaled nitric oxide (FeNO) was measured using an automatic apparatus (NIOX VERO, Circassia, Oxford, UK). During measurement, the participants had to exhale in the device for 6 s while the airflow and the expiration air volume should be kept constant and controlled by the apparatus. The FeNO in exhaled air was measured in parts per billion (ppb).

### 2.6. Near-Infrared Spectroscopy Measurement

The NIRS system (PortaMon, Artinis Medical Systems, Elst, Netherlands) was used to non-invasively assess sternocleidomastoid muscle oxygenation during the incremental resistive breathing protocol. Briefly, the NIRS probe is consisted of three optodes, emitting light at two wavelengths (760–850 mm), and one receiver, with an inter-optode distance of 30, 35 mm, and 40 mm. The NIRS probe was placed on the skin over the sternocleidomastoid muscle on the left body-side and fixed in position with optode holders covered in black tape to block exogenous light. The probe was always positioned by the same experimenter who ensured, as much as possible, the reproducibility of the placement. Since the NIRS device cannot discriminate between chromophores (i.e., hemoglobin and myoglobin in the muscle) and because myoglobin content tends to remain constant during exercise, the changes in NIRS signals can be attributed to changes in hemoglobin [33]. Thus, NIRS provided changes in the microvascular concentrations of oxyhemoglobin (Δ[O_2_Hb]) and de-oxyhemoglobin (Δ[HHb]), which reflect the dynamic balance between muscle oxygen delivery and extraction in the underlying tissue [36,37]. Moreover, total hemoglobin concentration (Δ[tHb]) was calculated as the sum of O_2_Hb and HHb. Of note, changes in tHb have been reported to reflect changes in microvascular blood volume [38]. Using spatially resolved spectroscopy, NIRS also calculated the tissue saturation index (TSI%), which reflects the balance between oxygen supply and demand, and is expressed as a percentage at absolute values [39]. The NIRS technique and the units used in the present investigation have been validated in research of the same nature [40,41]. Inspiratory muscle oxygenation was also evaluated at baseline, prior to (i.e., pre-resistive breathing) and after (i.e., recovery) the resistive breathing protocol, for 5 consecutive minutes. During the 5-min resting periods at baseline and before resistive breathing, the data from the last 30 s were averaged to obtain the baseline and pre-resistive breathing values, respectively. NIRS data were collected via Bluetooth at 10 Hz using Oxysoft software (Artinis Medical Systems, Elst, Netherlands) and the average of the three optodes was used for analysis.

### 2.7. Statistical Analysis

The distribution of all dependent variables was examined by the Kolmogorov–Smirnov test and was found not to differ significantly from normality. A two-way repeated-measures ANOVA test [(group (L-citrulline vs. placebo) × time (baseline, pre- resistive breathing, and post-resistive breathing or baseline, pre-resistive breathing, 70%, 80%, and 90% exercise intensity and recovery for the respiratory muscles resistive breathing)] was performed for pulmonary function indices, FeNO measurement, and maximal inspiratory pressure. When a significant interaction was obtained, pairwise comparisons were performed through the Sidak test that counteracts the problem of multiple comparisons. In none of the variables the assumption of sphericity was violated. Data are presented as mean  ±  standard deviation (SD), and the level of significance was set at a  =  0.05. The SPSS version 21.0 was used for all analyses (SPSS Inc., Chicago, IL, USA).

## 3. Results

### 3.1. Supplementation and Performance

**Figure 1:**
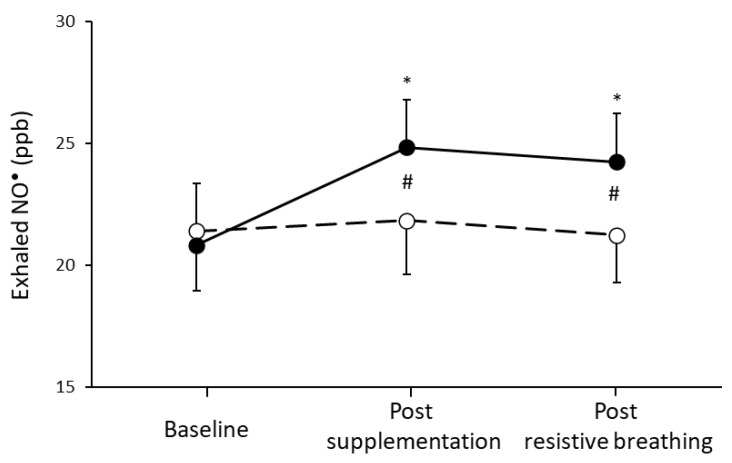
Exhaled NO^•^ at baseline, one hour post L-citrulline (closed circles) and placebo (open circles) supplementation and post resistive breathing to task failure. (*) indicates significant difference (*p* < 0.05) compared to baseline; (#) indicates significant difference (*p* < 0.05) between the L-citrulline and placebo conditions.

A significant condition by time interaction (*p* = 0.023) and a significant main effect of condition (*p* = 0.039) and time (*p* = 0.011) was found in exhaled NO^•^ concentration (Figure 1). Specifically, 1 hour post L-citrulline supplementation exhaled NO^•^ concentration was found to be increased, and this increase was preserved until the end of the incremental resistive breathing to task failure. On the contrary, no difference was observed in exhaled NO^•^ for the placebo condition at all time points of assessment.

**Figure 2:**
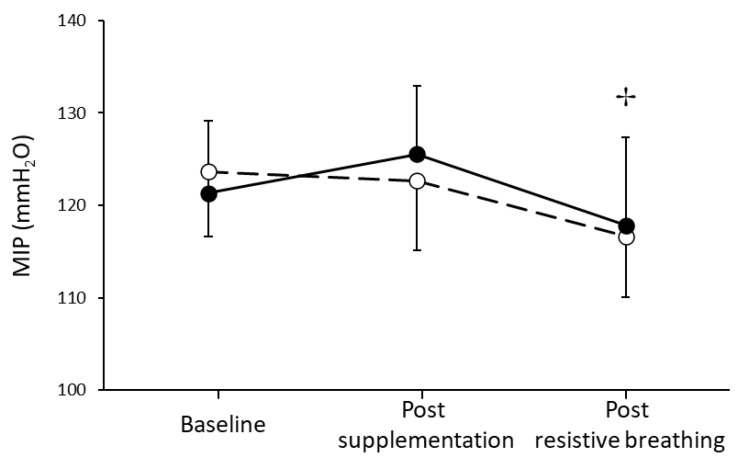
Maximal inspiratory pressure (MIP) at baseline, one hour post L-citrulline (closed circles) and placebo (open circles) supplementation and post resistive breathing to task failure. (✢) indicates significant main effect of time (*p* < 0.05).

Regarding MIP, there was no significant condition by time interaction (*p* = 0.956). However, a significant main effect of time was found for MIP (*p* = 0.047; Figure 2). MIP decreased −6.1% and −4.9% post resistive breathing for the L-citrulline and placebo groups, respectively, compared to the pre resistive breathing values. The L-citrulline supplementation and the concomitant NO^•^ increase did not improve performance since MIP declined similarly for both conditions during the incremental resistive breathing to task failure.

Additionally, during the last stage of the incremental resistive breathing (i.e., 90% of the maximal MIP), the number of breaths at task failure was not different between the L-citrulline and the placebo conditions (i.e., 41 ± 16 and 36 ± 15 number of breaths, respectively; *p* = 0.404).

There was a significant main effect of time for the Borg scale of perceived exertion. Specifically, for both conditions, the Borg scale score was significantly higher at the end of the resistive breathing session than baseline (i.e., 17.3 ± 1.6 and 16.8 ± 1.3 for L-citrulline and placebo, respectively; *p* < 0.001). However, neither a significant main effect of condition (*p* = 0.608) nor a condition by time integration (*p* = 0.521) was found between the L-citrulline and placebo conditions.

### 3.2. Respiratory Capacity and Muscle Oxygenation

**Figure 3:**
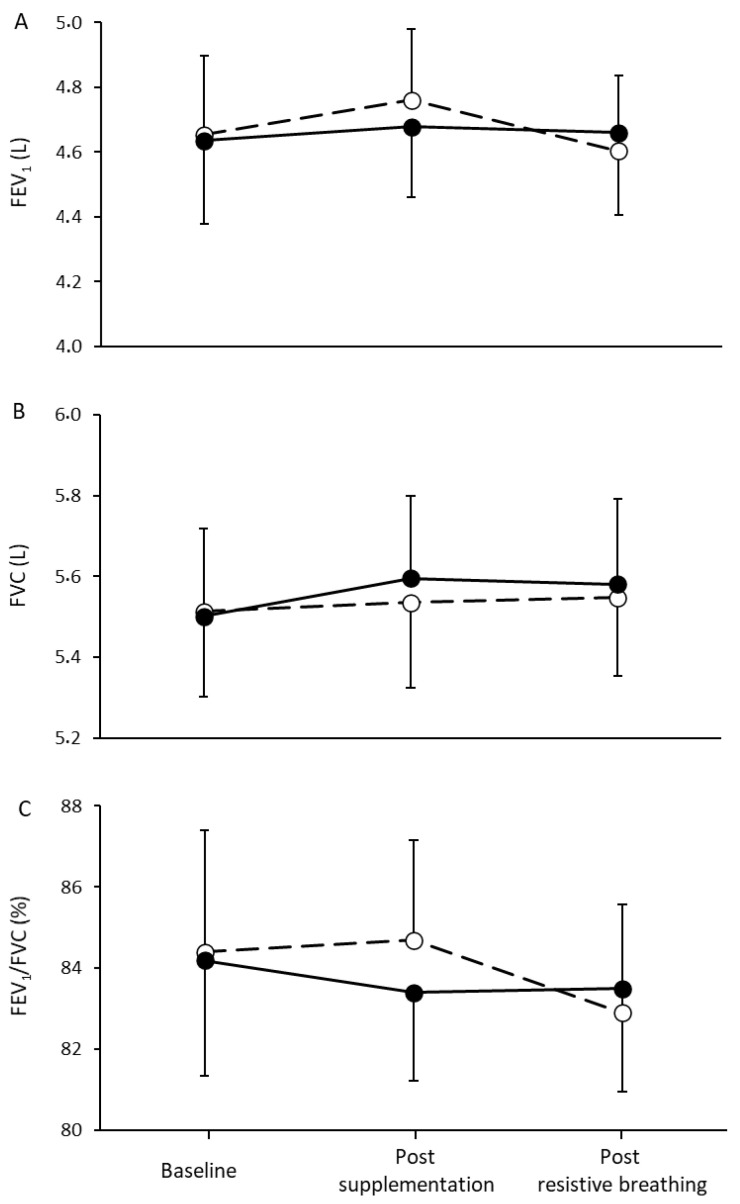
Forced expired volume in 1 second (**A**), forced vital capacity (**B**), and their ratio (**C**) at baseline, one hour post L-citrulline (closed circles) and placebo (open circles) supplementation and after resistive breathing to task failure.

No significant main effect of time and condition or a condition by time interaction was found during the evaluation of FEV_1_ (*p* = 0.598, *p* = 0.968, *p* = 0.713, respectively), FVC (*p* = 0.322, *p* = 0.926, *p* = 0.709, respectively), and FEV_1_/FVC (*p* = 0.627, *p* = 0.931, *p* = 0.736, respectively) (Figure 3). Specifically, FEV_1_, FVC, and their ratio were not affected either by the L-citrulline supplementation or after the end of the respiratory muscles resistive breathing to task failure (Figure 3A–C).

**Figure 4:**
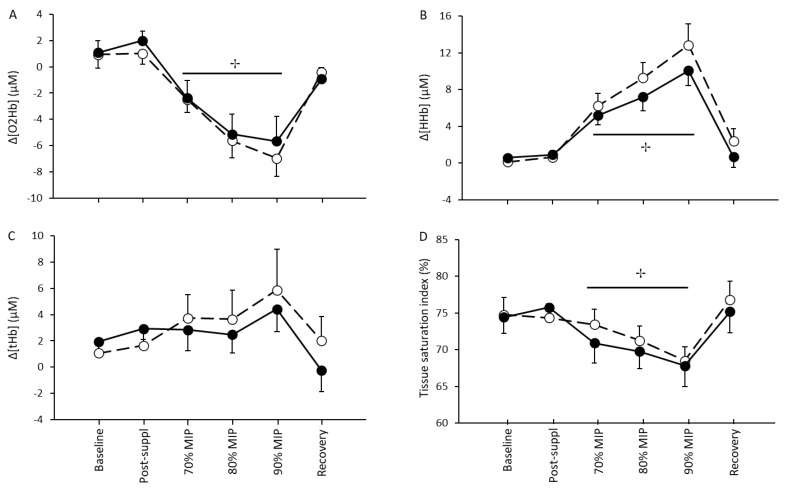
Sternocleidomastoid muscle oxyhemoglobin (O_2_Hb) (**A**), deoxyhemoglobin (HHb) (**B**), total hemoglobin (tHb) (**C**), and tissue saturation index at baseline, one hour after the L-citrulline (closed circles) and placebo (open circles) supplementation and after resistive breathing to task failure. (✢) indicates significant main effect of time (*p* < 0.05).

Regarding muscle oxygenation during the incremental resistive breathing to task failure, neither a main effect of condition nor a condition by time interaction was found in Δ[O_2_Hb] (*p* = 0.620 and *p* = 0.814, respectively), Δ[HHb] (*p* = 0.283 and *p* = 0.694, respectively), Δ[tHb] (*p* = 0.713 and *p* = 0.680, respectively) and TSI% (*p* = 0.770 and *p* = 0.591 respectively) between L-citrulline and placebo measured in sternocleidomastoid muscle (Figure 4A–D). However, a significant main effect of time was found for Δ[O_2_Hb] (*p* < 0.001), Δ[HHb] (*p* < 0.001), and TSI% (*p* < 0.001) during resistive breathing sessions. In particular, for both conditions, every resistive breathing intensity stage (i.e., 70%, 80%, and 90% of MIP) caused a significant decrease in Δ[O_2_Hb] and TSI% and a significant increase in Δ[HHb] compared to baseline. During the 5-min recovery period, all muscle oxygenation parameters returned to pre resistive breathing values.

## 4. Discussion

To the best of our knowledge, this is the first study examining the effect of acute L-citrulline supplementation on sternocleidomastoid muscle oxygenation and respiratory performance. We hypothesized that an increase in NO^•^ synthesis through L-citrulline supplementation would increase sternocleidomastoid muscle oxygenation and respiratory performance during an incremental resistive breathing test to task failure. However, the results of our investigation failed to support these hypotheses. Even though 6 g of acute L-citrulline supplementation significantly increased exhaled NO^•^.

### 4.1. L-Citrulline Supplementation and NO^•^ Bioavailability

NO^•^ has a vital role in regulating vasodilation, blood flow, and muscle oxygenation; thus, increasing NO^•^ bioavailability positively affects performance during exercise and recovery [13,42,43]. Consequently, supplementation with NO^•^ precursors such as L-citrulline and L-arginine to augment nitric oxide bioavailability and enhance performance is a common practice followed by athletes and physically active individuals. Of interest, L-citrulline supplementation appears to be more efficient in enhancing NO^•^ bioavailability compared to L-arginine because it bypasses hepatic metabolism, increasing this way the levels of extracellular L-arginine [3,5,17]. Indeed, previous studies reported that chronic L-citrulline supplementation might increase nitric oxide bioavailability [5,44]. In the present study, we found that a single dose of 6 g of L-citrulline increased exhaled NO^•^ one hour after the supplementation, which is supported by relevant studies that showed that after oral L-citrulline supplementation, L-arginine concentrations peak around one hour later [45,46] in a dose-dependent manner [5]. Thus, based on our results, a single dose of 6 g of L-citrulline supplementation 1 hour before exercise is sufficient for increasing NO^•^ bioavailability.

Certainly, considering the different origins of nitric oxide in skeletal muscle (i.e., neuronal, and endothelial) and exhaled air (i.e., epithelial) [47], some plasma or skeletal muscle measurements of nitric oxide production and/or metabolism could have added insightful mechanistic information. In the present study, our purpose was to examine whether acute L-citrulline supplementation would affect sternocleidomastoid muscle blood flow, oxygenation, and respiratory performance. Thus, the lack of any NO^•^ measurements in skeletal muscle is a significant limitation of the present study. Yet, we have particularly focused on the respiratory system and pulmonary function, and have chosen, therefore, nitric oxide in exhaled air. Additionally, blood pressure and heart rate after L-citrulline supplementation were not measured since changes in these parameters were observed after chronic L-citrulline supplementation [48]. Moreover, alterations in blood pressure after L-citrulline supplementation were observed mainly in participants with health issues. Such as obese postmenopausal women [49,50], elderly individuals [32], and heart failure patients [51], while in the present investigation, young, healthy individuals were recruited.

### 4.2. Inspiratory Muscle Performance and Resistance to Fatigue

Inspiratory muscle dysfunction is a primary contributor to ventilatory failure during fatiguing conditions such as exercise [52]. During intense exercise, inspiratory dysfunction can occur due to increased work of breathing and/or insufficient blood flow and oxygen delivery to the respiratory muscles, progressively leading to fatigue and impairing exercise performance [25]. The sternocleidomastoid muscle is a crucial accessory muscle [53] that is highly active during exercise, supporting the primary inspiratory muscles’ work [54]. Additionally, sternocleidomastoid muscle deoxygenation has been reported to be progressively increased during incremental inspiratory loading [55]. Thus, it was hypothesized that enhancing sternocleidomastoid muscle blood flow and oxygen delivery through L-citrulline supplementation might favorably affect respiratory muscle performance and resistance to fatigue during incremental resistive breathing.

However, contrary to our hypothesis, acute L-citrulline supplementation and the concomitant NO^•^ increase did not improve sternocleidomastoid muscle performance and resistance to fatigue either one hour post supplementation or post-respiratory muscles resistive breathing to task failure. Comparable results have been reported in studies of the same nature using clinical and healthy populations [56,57]. However, in these investigations, the supplementation was L-arginine. L-arginine can be metabolized in the liver, contrary to L-citrulline, which mainly contributes to NO^•^ production [3,17]. Specifically, MIP and the number of breaths at exhaustion were not different between the L-citrulline and the placebo conditions. Additionally, FEV_1_, FVC, and their ratio were not affected by the L-citrulline supplementation. Even though our resistive breathing to task failure protocol induced fatigue, perhaps it was insufficient to cause extensive disturbances on respiratory performance, as observed in other studies using resistive breathing [58,59,60]. Given the fact that sub-maximal constant endurance exercise induces significant fatigue in respiratory muscles [61], it could be suggested that future studies examining inspiratory muscle fatigue should employ constant whole-body high-intensity endurance exercise protocols to compromise blood flow to inspiratory muscles and induce fatigue. Indeed, during whole-body exercise, the manipulation of inspiratory muscle work with resistors will increase the competitiveness between inspiratory and locomotor muscles for blood flow and oxygenation [23,62]. Thus, we believe that it is more likely to find any favorable effect of a supplement on fatigue when the exercise protocol involves whole-body exercise and greater fatigue levels occur in the muscle under examination.

### 4.3. Sternocleidomastoid Muscle Oxygenation

In the present study, we used NIRS that continuously monitors regional tissue oxygenation in vivo. We found that after both resistive breathing conditions (i.e., L-citrulline or placebo supplementation) and at every intensity stage (i.e., 70%, 80%, and 90% of MIP), there were a significant decrease in Δ[O_2_Hb] and TSI% and a significant increase in Δ[HHb] compared to baseline in the sternocleidomastoid muscle, which is in line with previous studies [55]. The muscle’s oxygenation and deoxygenation responses during loading were as expected in order to facilitate oxygen supply to the working muscles. However, despite the changes we observed after incremental inspiratory muscles resistive breathing to task failure in these parameters, L-citrulline supplementation did not affect sternocleidomastoid muscle oxygenation. Considering the role that NO^•^ has in blood flow and the increase we observed after L-citrulline supplementation in exhaled NO^•^, we expected that L-citrulline supplementation would have improved sternocleidomastoid muscle oxygenation.

Similar results were reported in a study where acute L-citrulline supplementation enhances NO^•^ bioavailability but had no effect on blood flow in young and older adults [63]. Additionally, no effect on blood flow was observed after ingestion of a combination of 10 g of L-citrulline with whey protein in older adults [64]. On the contrary, after longer-term supplementation with concentrate watermelon juice (providing 3.4 g/day of L-citrulline for 16 days), the tissue oxygenation index of the vastus lateralis was enhanced during moderate-intensity exercise [44]. Similarly, supplementation with L-citrulline (6 g/day for 7 days) increased VO_2_ uptake kinetics of the vastus lateralis during moderate and high-intensity exercise in recreationally active adults [16]. Furthermore, muscle blood flow during exercise was improved after 14 days of L-citrulline supplementation (6 g/day) in older men [31]. Therefore, a chronic supplementation intervention could be required to successfully improve inspiratory muscle oxygenation and resistance to fatigue.

## 5. Conclusions

In the present study, we found that a single ingestion of 6 g of L-citrulline 1 hour before resistive breathing significantly increased NO^•^ bioavailability. Considering the strong ergogenic effects that NO^•^ has on exercise performance, this observation is of utmost importance for acute L-citrulline ingestion 1 hour before exercise events. However, neither respiratory muscle performance and resistance to fatigue nor sternocleidomastoid muscle oxygenation and deoxygenation responses were enhanced. These results imply that other reasons than NO^•^ bioavailability might affect sternocleidomastoid muscle performance and blood flow during resistive breathing to task failure of the inspiratory muscles. In our opinion, L-citrulline supplementation is worthy of further consideration from the scientific community, especially in patients with cardiovascular or pulmonary diseases (e.g., lower-extremity artery disease, chronic obstructive pulmonary disease) that are usually characterized by insufficiently blood flow and oxygen delivery to the muscles. It would also be interesting for future studies to examine the effect of short-term (e.g., 7-day) supplementation with L-citrulline on inspiratory muscles performance and oxygenation.

## Data Availability

Data from the current study are available from the corresponding author upon reasonable request.
